# Why Temporal Persistence of Biometric Features, as Assessed by the Intraclass Correlation Coefficient, Is So Valuable for Classification Performance

**DOI:** 10.3390/s20164555

**Published:** 2020-08-14

**Authors:** Lee Friedman, Hal S. Stern, Larry R. Price, Oleg V. Komogortsev

**Affiliations:** 1 Department of Computer Science, Texas State University, 601 University Dr, San Marcos, TX 78666, USA; ok11@txstate.edu; 2 Department of Statistics, University of California, Irvine, CA 92697, USA; hal.stern@uci.edu; 3 Methodology, Measurement & Statistics Office of Research & Sponsored Programs, Texas State University, 601 University Dr, San Marcos, TX 78666, USA; lp11@txstate.edu

**Keywords:** biometrics performance, temporal persistence, normally distributed features

## Abstract

It is generally accepted that relatively more permanent (i.e., more temporally persistent) traits are more valuable for biometric performance than less permanent traits. Although this finding is intuitive, there is no current work identifying exactly where in the biometric analysis temporal persistence makes a difference. In this paper, we answer this question. In a recent report, we introduced the intraclass correlation coefficient (ICC) as an index of temporal persistence for such features. Here, we present a novel approach using synthetic features to study which aspects of a biometric identification study are influenced by the temporal persistence of features. What we show is that using more temporally persistent features produces effects on the similarity score distributions that explain why this quality is so key to biometric performance. The results identified with the synthetic data are largely reinforced by an analysis of two datasets, one based on eye-movements and one based on gait. There was one difference between the synthetic and real data, related to the intercorrelation of features in real data. Removing these intercorrelations for real datasets with a decorrelation step produced results which were very similar to that obtained with synthetic features.

## 1. Introduction

In a recent report [1], we introduced the intraclass correlation coefficient (ICC) as an index of temporal persistence (e.g., stability, permanence) of single biometric features. In that report and the present report, we are exclusively dealing with features which are normally distributed or can be transformed into a normal form. (We understand that this limitation would mean that this analysis might not be directly applicable to several important biometric modalities, including fingerprints and iris scans. Other modalities such as face recognition can meet these criteria [2]. In the present report we apply these concepts to eye movements and gait. The analysis also applies to brain structure [1]. We think that this approach will be applicable to most physiological (EEG, ECG, etc.) and behavioral modalities.) The ICC can only be calculated if each subject is tested on 2 or more occasions. For a biometric system, with multiple features available for selection, the ICC can be used to measure the relative stability of each feature. In the report, we also showed that choosing only the most temporally persistent features yielded superior performance in 12 of 14 datasets (*p* = 0.0065, one-tailed). Thirteen of the 14 datasets in that paper were real datasets from a number of different biometric modalities, including oculomotor, face, gait and brain structure. In general, then, for those datasets, prescreening potential biometric features, and choosing only higy reliable features yielded better performance than choosing lower ICC features or than choosing all features combined. For eye movement-driven biometrics, the use of reliable features, as measured by ICC, allowed to us achieve an equal error rate (EER) of 2.0% (Equal error rate (EER) is the rate at which both acceptance and rejection errors are equal. The value of the EER can be easily obtained from the ROC curve. The EER is a quick way to compare the accuracy of devices with different ROC curves. In general, the device with the lowest EER is the most accurate. (https://en.wikipedia.org/wiki/Biometrics)), which was not possible before. In that report [1], we did make some attempt to answer the question addressed by this manuscript (see Section 2.2 for details). We present this report to more fully, accurately and precisely answer the question posed by this manuscript. In the service of this goal, we present here a method for creating synthetic datasets with a number of properties that are helpful for studying biometric performance. Since the data are synthetic, we are able to control the degree of temporal persistence of the features while also ensuring that features are approximately independent of each other and thus provide unique pieces of information for biometric verification. We think that having unique pieces of information will allow us to address several theoretical notions relevant to biometric analysis in this and subsequent studies. In addition to the present manuscript, we have prepared two other manuscripts on unrelated topics which employ these synthetic features [2,3]. Therefore, assuming their acceptance by at least some of the research community, these synthetic features may provide a useful tool for additional biometric research.

Section 2 reviews the relevant literature. In Section 3, we present our method to create synthetic datasets and show the theoretical relationship between temporal persistence and the distributions of similarity scores that are used in biometric systems (Experiment 1). Section 4 and Section 5 explore two biometric datasets, one based on eye-movement features (Experiment 2), and the other based on gait-related features (Experiment 3). In Section 6, we identify one aspect of the synthetic data that differs from the real data and investigate the impact of this difference on biometric performance. Section 7 provides a closing discussion.

## 2. Prior Work

### 2.1. Prior Work on Permanence in Biometrics

There are many reports which assess either template aging or permanence that operate at the level of a complete biometric system rather than at the level of single features [4,5,6,7,8,9,10,11,12,13,14,15,16,17,18] which is our interest here. For example, one recent paper [19] proposes a new method for measuring biometric permanence. Although these authors state that their method provides estimates of the permanence of biometric features, their measure of permanence is actually at the level of a complete biometric system rather than at the level of individual features. This is also true for the report on the permanence of ECG biometrics [20], where permanence is assessed by looking at plots of EER over various time intervals.

We have carefully reviewed a number of the references from the template aging literature. These tend to provide evidence that the performance of entire biometric systems degrades over time. For us, this still leaves unanswered the key question of “which features of these systems are changing over time?” We think that the ICC, or similar index, can quantify the relative permanence or feature stability for individual features over different time intervals. Once researchers in the field of template aging have this kind of feature level data, new analyses and steps to reduce template aging are possible. For example, one can pinpoint the specific features that are creating the problem. Then these features can be investigated more deeply. Perhaps there is some way to increase the permanence of these individual features? Perhaps there is some way to choose or design similar features with greater permanence? Perhaps individual features should be dropped from the analysis and replaced by more permanent features?

Therefore, we believe our interest in feature permanence and its potential role in feature selection and improved biometric performance offers potential benefits to the template aging literature. Specifically, researchers who want to reduce template aging effects, can benefit from our approach.

Jain [21] discusses the importance of the permanence of biometric features but does not provide a method for assessing the permanence of individual features. In our prior paper [1], we introduced the ICC for the assessment of the persistence of individual biometric features. Another recent paper [22] creates indices of permanence for brain waves. This method is specific for this modality, or at least for features which emerge from time-series (perhaps ECG, for example).

A concept related to temporal persistence is that of “reliable bits” in binary or quantized biometric features, as it tries to identify those bits in biometric features that show minimal intra-subject variation. We may assume that these bits will also be temporally persistent as long as the features themselves do not change with time. The idea of identifying reliable bits was first introduced in [23] for binarized features derived from fingerprint templates. Here only features were binarized (quantized to 1 bit) that had a certain distance to the binarization threshold. In [24,25] more advanced methods to extract reliable bits from quantized features were proposed, in which the number of quantization levels per feature was chosen such that recognition performance is optimized, given a maximum total number of bits to encode the features. The optimization assumes Gaussian probability density functions (PDFs) for the features, and turns out to work well in practice. In [23,24,25], additional information has to be added to the biometric template that indicates how features are quantized.

In terms of accuracy, we expect that the use of reliable bits as presented in [23] in a metric for temporally persistence could result in at most similar performance as that of ICC, because the features are coarsely quantized to 1 bit. The reliable bits according to [24,25] might have similar performance to the ICC as they are the result of a finer quantization. Further, empirical, research is needed to confirm these expectations. However, the computation of reliable bits according to [24,25] requires a constraint optimization that is more computationally demanding than the computation of ICC.

### 2.2. How This Work Differs from Our Earlier Article

In our prior report [1], we introduced the concept of the interclass correlation coefficient as a method to assess the temporal persistence (or permanence) of each individual biometric feature. We showed that using only the most temporally persistent features produced markedly improved biometric performance. These are not the goals of the present manuscript.

The goal of the present manuscript is to inquire why temporal persistence is so effective in improving biometic performance. We did partly address this question in our earlier manuscript. In that case, the level of analysis was between biometric datasets. In the present case, we are looking at the performance of different sets of features within a biometric dataset. The prior analysis is complicated and confounded by differences between datasets. Datasets can differ in modality, number of features, intercorrelation of features, number of subjects, overall performance, etc. Comparisons within a dataset are not complicated or confounded by these more general differences. This difference between our earlier analysis and the present analysis is similar to the difference between “between-subjects” analyses and “within subjects” analyses in statistics. In within-subjects designs:

…“each subject (*dataset*) serves as his or her (*its*) own control. This typically gives within-subjects designs considerably more power than between-subjects designs. That is, this makes within-subjects designs more able to detect an effect of the independent variable than are between-subjects designs.” (online statistics book: http://onlinestatbook.com/2/research_design/designs.html#:~:text=An%20advantage%20of%20within%2Dsubjects,levels%20of%20performance%20are%20controlled.&text=That%20is%2C%20this%20makes%20within,than%20are%20between%2Dsubjects%20designs).

## 3. Creation and Analysis of Synthetic Datasets

### 3.1. Creation of Synthetic Data

Recall that the intraclass correlation coefficient (ICC) is a measure of the correlation expected for repeated measurements of the same feature on different occasions. Unlike the Pearson r correlation coefficient, which is typically applied as an interclass measure of relative agreement (i.e., two series can be correlated even if they differ substantially in level and spread), the ICC is an intraclass measure of absolute agreement [1]. Measures from the same set of subjects at two different times are intraclass measurements (same metric and variance). ICC ranges from 0.0 to 1.0 with the latter corresponding to perfect temporal persistence. Our goal is to create synthetic features with a specified target ICC (denoted $ICC_{Target}$). Let $X_{ijs}$ denote the measurement of feature *j* (j=1,⋯,K) on session (occasion) *s* (s=1,⋯,S) for individual *i* (i=1,⋯,N). Although the ICC can be calculated based on many sessions, in our experience, biometric assessment is typically performed comparing only two points in time. Therefore, henceforth we will set S=2. We generate normally distributed features such that the theoretical intraclass correlation of repeated measurements of the same feature on the same subject is $ICC_{Target}$ while the theoretical correlation of measurements of different features on the same individual and the theoretical correlation of measurements from different individuals are zero. In practice when data are simulated there are small variations in the empirical ICCs and there are small intercorrelations between features (and individuals) due to chance. Code (“r”) to create datasets of synthetic features is included as Appendix A.

**Figure 1:**
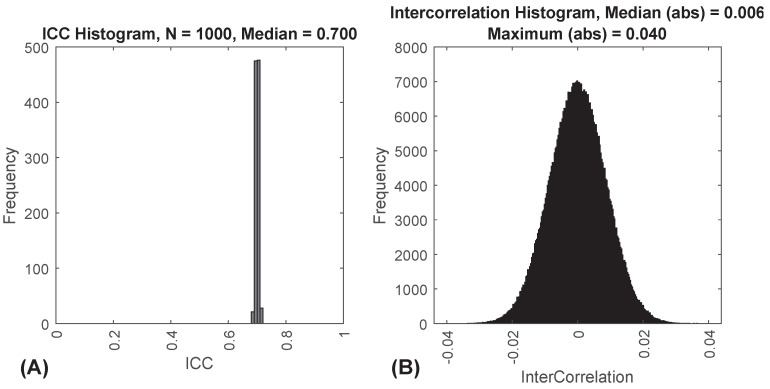
(**A**) Frequency histogram of intraclass correlation coefficients (ICCs) for 1000 features with an $ICC_{Target}=0.7$. This is from a synthetic dataset with 10,000 subjects. (**B**) Frequency histogram of correlations between 1000 features for 10,000 subjects, two sessions, with an $ICC_{Target}=0.7$. Note that the median and maximum are of the absolute value of the correlations.

Using this method, we can create features which are normally distributed, that have specified ICCs, with as many subjects and sessions as we desire. These features all have mean = 0 and SD = 1. These features are generally independent, but there are some small intercorrelations between features due to chance. To illustrate the approach, we generated data for 10,000 subjects, 1000 features and 2 occasions with $ICC_{Target}=0.7$. Figure 1A shows a histogram of the resulting empirical ICCs. Figure 1B shows a histogram of the resulting inter-feature correlations.

### 3.2. Creation of Sets of Features with Varying Degrees of Persistence

To study the relationship of temporal persistence and biometric performance we generate a series of synthetic datasets with varying ICCs. To be specific we create 10 different datasets with each dataset consisting of 50 features and the features in each dataset having ICC values that vary over a small interval (e.g., 0.6 to 0.7). We denote the datasets as “Bands” to indicate that they cover different bands of the range of possible ICCs. Band 0 consists of 50 features simulated to have ICCs between 0.0 and 0.1 (with 5 features generated using Algorithm 1 with $ICC_{Target}=0.005$, 5 features generated with $ICC_{Target}=0.015$, …, 5 features generated with $ICC_{Target}=0.095$). Band 1 has ICCs between 0.1 and 0.2 (again evenly spread out across that range), and so on through Band 9 which has ICCs between 0.9 and 1.0.
**Algorithm 1** Creating Synthetic Features.** Input**: *N* (subjects), *K* (features), $ICC_{Target}$ ** Output**: 3-dimensional (N×K×2) feature matrix $X_{ijs}$ with desired correlation structure for j=1,⋯K  for i=1,⋯N   Set $X_{ij1}=Z$ where *Z* is a random standard normal deviate.   Set $X_{ij2}=X_{ij1}$ for j=1,⋯K  for i=1,⋯N   for s=1,2    Set $X_{ijs}=X_{ijs}+W$; where *W* is a random normal deviate with mean = 0 and     standard deviation = $\sqrt{\left(1-ICC_{Target}\right)/ICC_{Target}}$ For each feature *j*, treat $X_{ijs}$ as a single vector of length N·S and apply a z-score transform to ensure mean = 0 and standard deviation = 1

### 3.3. Biometric Performance Assessment

For each band (dataset), distance scores were calculated using only 20 randomly chosen features from the full set of 50 features in each band. We chose this number empirically based on the range of EER values produced across the bands. We employed the cosine distance metric, since we have shown in an earlier (unpublished) report that the best biometric performance is produced with this choice (https://www.doi.org/10.13140/RG.2.2.17510.06727). The resulting distance measures were scaled to go from 0 to 1 and then they were reflected (1– distance) to compute similarity scores. A “genuine” distribution of similarity scores was constructed from the similarity scores for each subject and his/her self. All other similarity scores were considered impostors. These data were submitted to a ROC analysis and the EER was computed.

### 3.4. Plotting Similarity Score Distributions for Each Band

**Figure 2:**
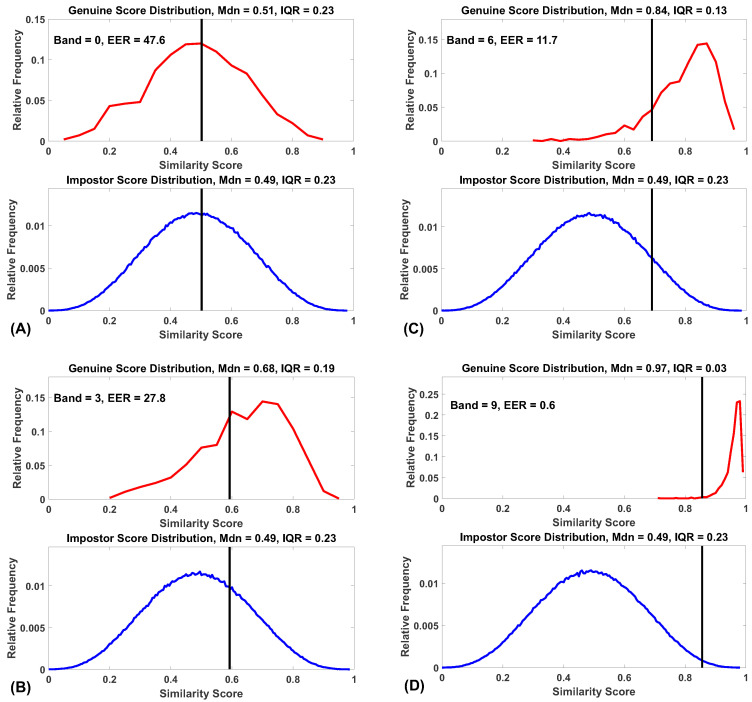
Similarity score distributions for Bands 0, 3, 6 and 9. (**A**) Distributions for Band 0 (ICC from 0.0 to 0.1). (**B**) Distributions for Band 3 (ICC from 0.3 to 0.4). (**C**) Distributions for Band 6 (ICC from 0.6 to 0.7). (**D**) Distributions for Band 9 (ICC from 0.9 to 1.0). Frequency is expressed as a proportion of all values. Mdn = median, IQR = interquartile range. Vertical black lines represent the threshold at which EER was achieved. For each band, 20 features were chosen randomly.

**Figure 3:**
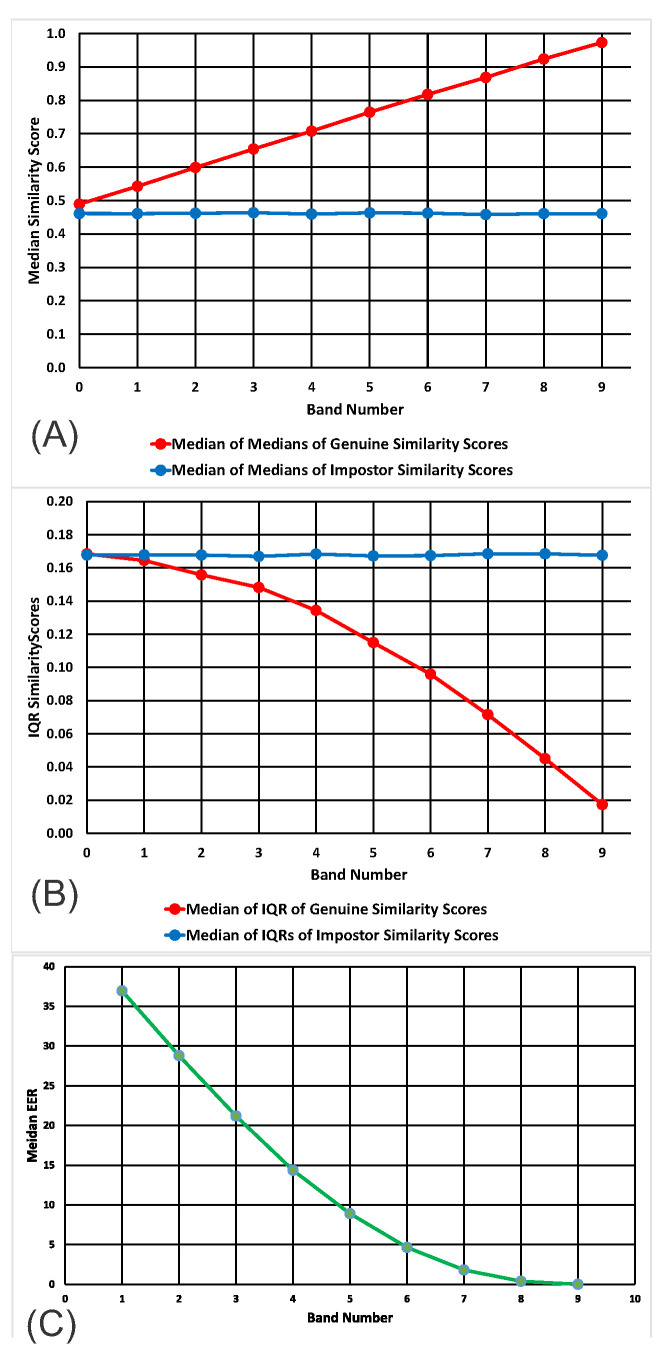
Medians and IQRs of genuine and impostor similarity scores and EERs across bands. (**A**) Medians for synthetic data. Each point is the median of 10 median estimates. (**B**) IQRs for synthetic data. Each point is the median of 10 IQR estimates. (**C**) Median EERs. Ranges for the values in this figure can be found in Appendix B, Table A1, Table A2 and Table A3.

After each ROC analysis for each band, we displayed the similarity score distributions, as in Figure 2. For each band, 20 features were chosen randomly. From inspection of these, it became clear that the distributions of imposter similarity scores were not changing as we moved from Band 0 to Band 9, but that there were marked changes in the distributions of genuine similarity scores. The medians of the genuine distributions increase from Band 0 to Band 9, and the interquartile ranges (IQR) of the genuine distributions decrease from Band 0 to Band 9. These patterns are clearly shown in Figure 2 and Figure 3.

### 3.5. Discussion of Results

The main findings of Experiment 1 are that using synthetic features with higher temporal persistence for biometric analysis produces lower EER values, i.e., improved biometric performance. These improvements are due to an increased median and a decreased IQR of the genuine similarity score distributions with no change in the impostor distributions. These results explain why features with increased temporal persistence produce better biometric results.

## 4. Evaluation of the SBA Dataset

### 4.1. Description of the SBA Dataset

The real eye movement data we employed for this study came from 298 subjects recorded on two sessions. For more details, see [1]. Subjects in the original study viewed 7 different tracking tasks. Only the text-reading task is relevant for the current report. Each subject was asked to read, silently, an identical pair of quatrains from the famous nonsense poem, “Hunting for a Snark”, written by Lewis Carroll (written from 1874–1876). The EyeLink 1000 (SR Research Ltd., Kanata, ON, Canada), a video-oculography system which employs detection of both the pupil and the corneal reflection to determine gaze position, was used to record eye movements. It records both vertical and horizontal eye movements. In the present study, only left eye movements were collected. For 298 subjects, we have a mean spatial accuracy of 0.50 degrees of visual angle (SD = 0.17, min = 0.20, max = 1.06). For further specifications, see the SR-Research website (https://www.sr-research.com/). The sampling rate for our data was 1000 Hz. Custom software transformed the raw records into gaze position data, in visual angle units, using the calibration data collected at the start of each task. The Stampe heuristic spike removal algorithm was employed [26]. In addition, blinks were detected and removed from the data by the EyeLink 1000. The eye movements were analyzed off-line. On each subject visit, subjects were studied twice (Sessions 1 and 2), approximately 20 min apart. They were given 60 s to read the poem passage. Session 1 to Session 2 (task-to-task) time intervals ranged from 13 min to 42 min (mean = 19.5; SD = 4.2). For eye movement classification, we employed the MNH method described in [27]. It identifies fixation periods, saccades and post-saccadic oscillations (PSOs) as well as periods of artifact and noise. Other portions of the recordings were left unclassified. For details regarding feature extraction, see [28].

### 4.2. Biometric Assessment of SBA Dataset

The ICCs for the SBA dataset were available from [1]. Our goal was to divide up the data into “temporal persistence” bands with equal numbers of features in each band. We found that if we created band limits based on ICCs from 0.0 to 0.9 in steps of 0.1, we would have at least 19 features per band.

For each dataset, standard ROC analyses were performed and similarity score distribution characteristics (median and IQR) were saved and plotted as a function of band number.

### 4.3. Results with SBA Dataset

**Figure 4:**
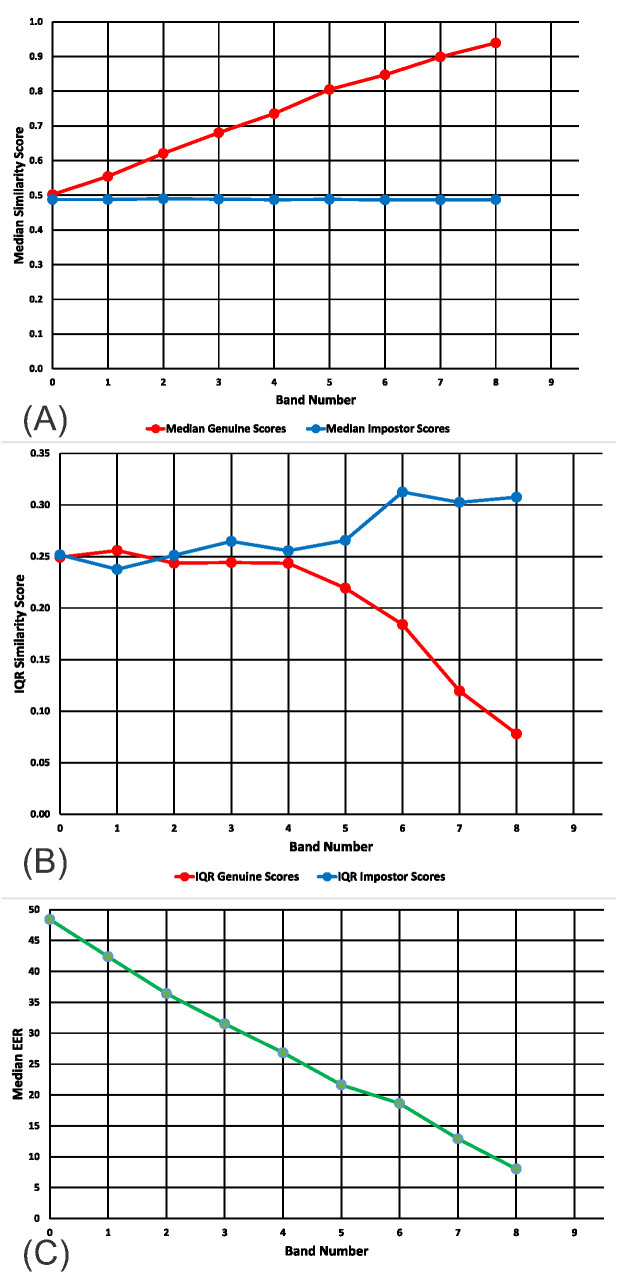
Medians and IQRs of genuine and impostor similarity scores across bands. (**A**) Medians for SBA real dataset. (**B**) IQRs for SBA real dataset. (**C**) Median EERs. Ranges for the values in this figure can be found in Appendix B, Table A3, Table A4 and Table A5.

In Figure 4A, we present the medians for the genuine and impostor distributions, for bands 0 (ICCs: 0.0 to 0.1) to 8 (ICCS: 0.8 to 0.9) for the real SBA dataset. In Figure 4B, we present the interquartile ranges for the genuine and impostor distributions, for bands 0 to 8 for the real SBA dataset.

## 5. Evaluation of the Gait1 Dataset

### 5.1. Description of the Gait1 Dataset

The Gait1 database is based on the Southampton Large Population Gait database of gait-related images, and videos [29]. These databases are comprised of over 100 subjects tested on many sessions. Sessions can be as little as 1 min apart. The analysis starts with a series of image frames while a subject walked. Binary silhouettes of the walking human were created [30]. The silhouette extraction used chroma-key subtraction in conjunction with a connected components algorithm. The silhouettes were resized to 64 × 64 pixels.

A series of image masks (NMsk masks), like a mask for a horizontal band near the subject’s waist, or the upper half of the silhouette, were applied to each subject silhouette, for each consecutive frame and a time series for each mask type for each subject for each session was produced. At this stage, each subject was characterized by the time series of NMsk masks. A cubic spline curve was fitted for the whole gait cycle, and 30 evenly spaced samples were taken from the whole curve, giving a single vector for each area mask used. These multiple vectors, for each 1 to NMsk mask, were reduced to a fewer dimensions, using canonical analysis. For each subject, the first feature was the first value in the final single vector, the second feature was the second value in this vector and so on.

### 5.2. Biometric Assessment of the Gait1 Dataset

The ICCs for the Gait1 dataset were also available from [1]. Once again, our goal was to divide up the data into “temporal persistence” bands with equal numbers of features in each band. We found that if we created band limits based on ICCs from 0.4 to 0.8 in steps of 0.1, we would have at least 7 features per band. There were simply too few features with lower ICCs to create reasonable bands. We randomly chose 5 of the 7 available features 10 times. Standard ROC analyses were performed on each dataset and similarity score distribution characteristics (median and IQR) were saved and plotted as a function of band number.

### 5.3. Results with Gait1 Dataset

**Figure 5:**
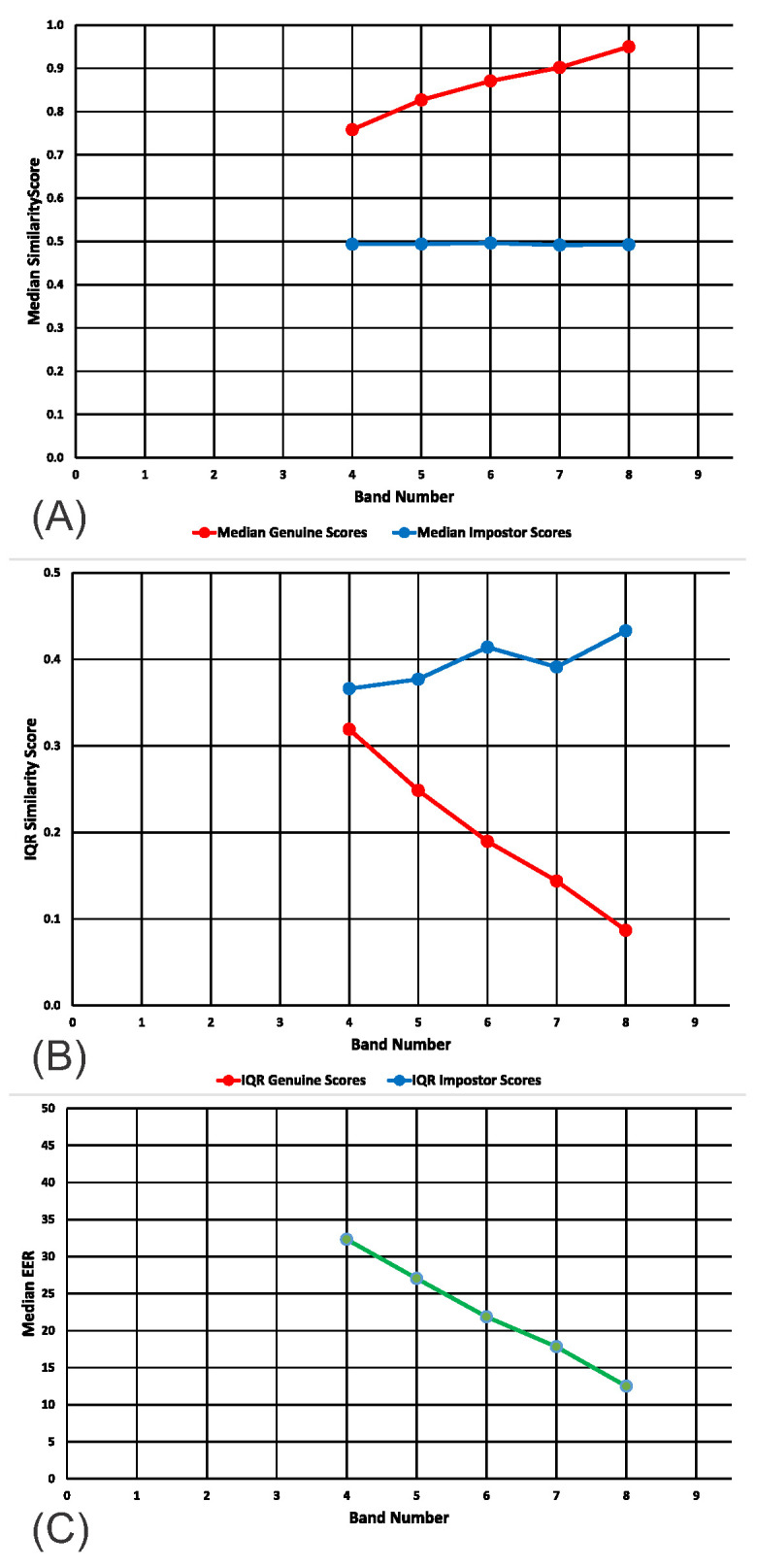
Medians and IQRs of genuine and impostor similarity scores across bands. (**A**) Medians for Gait1 real dataset. (**B**) IQRs for Gait1 real dataset. (**C**) Median EERs. Ranges for the values in this figure can be found in Appendix B, Table A3, Table A6 and Table A7.

In Figure 5A, we present the medians for the genuine and impostor distributions, for bands 4 (ICCs: 0.4 to 0.5) to 8 (ICCS: 0.8 to 0.9) for the real Gait1 dataset. In Figure 5B, we present the interquartile ranges for the genuine and impostor distributions, for bands 4 to 8 for the real Gait1 dataset.

## 6. Comparing Synthetic and Real Data Results

### 6.1. Discussion of Results

For both the SBA and the Gait1 datasets, the pattern of results was similar to that obtained from the synthetic data. In all cases, the median of the distribution of genuine similarity scores increased linearly with ICC Band (Figure 3A, Figure 4A and Figure 5A, red lines). In all cases, the median of the distribution of imposter similarity scores was mostly flat and unrelated to ICC Band (Figure 3A, Figure 4A and Figure 5A, blue lines). In all cases, the IQR of the distribution of genuine similarity scores decreased with increasing ICC Band (Figure 3B, Figure 4B and Figure 5B, red lines). However, there was one obvious difference between the results for the datasets and the results for synthetic features. For our synthetic data, the IQR of the distribution of imposter scores was flat and unrelated to ICC band whereas for both real feature datasets, there was an increase in the IQR of the distribution of imposter scores as the ICCs increased (Figure 3B, Figure 4B and Figure 5B, blue lines).

To explain the observed differences in the behavior of the IQR of the distribution of imposter scores, we focus on one key difference between the synthetic data and the real data. The synthetic features were generally uncorrelated (up to simulation error), whereas within real datasets, the features would be intercorrelated. The degree of intercorrelation of real features increases with ICC, since high ICC features are less noisy than low ICC features. (The ICC is the ratio of subject variance to the sum of subject variance, session variance and error variance. As a general matter, high ICC features will have lower error variance than low ICC features.) Below, we report the results of a series of analyses investigating if this increasing intercorrelation could explain the differences noted between the biometric results using synthetic data and real data.

### 6.2. Relationship between ICC Band and Median Intercorrelation between Features

**Figure 6:**
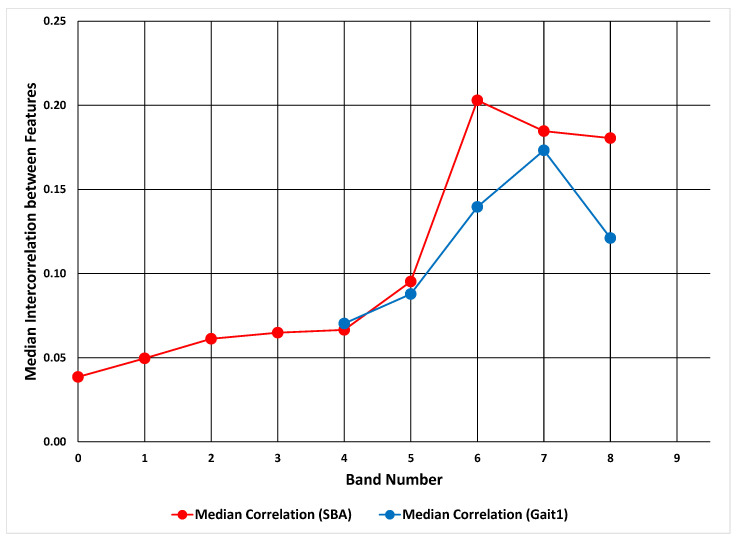
Relationship between ICC band and median intercorrelation between features. The results for the SBA dataset are presented in red and the results for the Gait1 dataset are presented in blue.

For both real datasets, the median intercorrelation (absolute values) increased with increasing ICC band (Figure 6). From this figure we can see that, for both datasets, there is a trend toward higher intercorrelation with increasing ICC band. It is not monotonic, but the increase is still obvious.

### 6.3. Relationship between the IQR of Impostor Distributions and Median Intercorrelation

**Figure 7:**
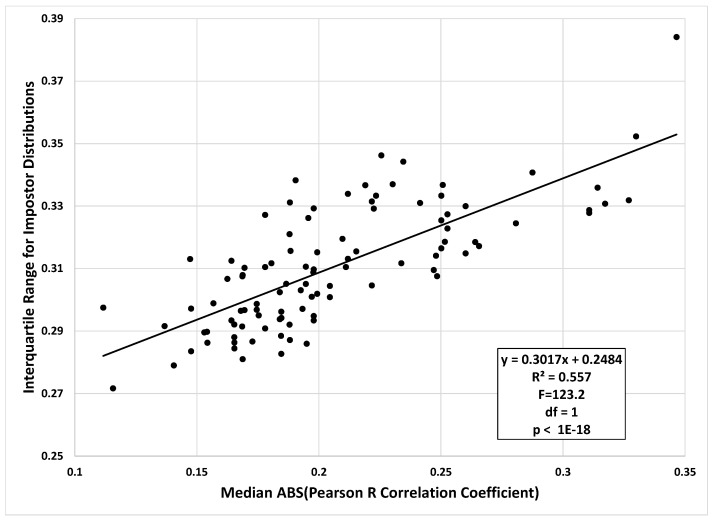
Relationship between IQR of the impostor distribution and median Pearson r correlation between features. As noted in text, the data are from the SBA real dataset.

To test if the IQR of the impostor distribution was related to the median intercorrelation between features in a dataset, we first combined the features from SBA Band 6 and Band 7. This yielded a combined dataset with 38 features. For 100 iterations, we randomly sampled, without replacement, 10 of the 38 features, computed the median Pearson r correlation coefficient (absolute value), and also computed the IQR of the impostor similarity scores for the random subset of features (Figure 7). From this figure we can see that the IQR of the impostor distribution increased linearly with increasing intercorrelation among features.

### 6.4. The Effects of Decorrelation

As a result of the analysis of the raw real data for the SBA dataset in Figure 4, we were interested in comparing the raw data with a decorrelated version of the data. If *N* = number of subjects, *S* = number of sessions and *K* = number of features, then we collect the data in a matrix *X* with *K* columns and r=N·S rows. Let $Z_{r,r}$ be an *r* by *r* square matrix with every element 1. Define

(1) $$D=X-\left(\frac{1}{r}\right)Z_{r,r}X$$

which is the data transformed so every random variable has zero mean. Then

(2) $$T=D{\left(D^{T}D\right)}^{-\frac{1}{2}},$$

where the exponent of −1/2 represents the matrix square root of the inverse of a matrix, is a matrix of *K* columns and *r* rows, where all of the columns are completely uncorrelated. This is known as the inverse Cholesky factorization [31]. (See also: https://blogs.sas.com/content/iml/2012/02/08/use-the-cholesky-transformation-to-correlate-and-uncorrelate-variables.html).

**Figure 8:**
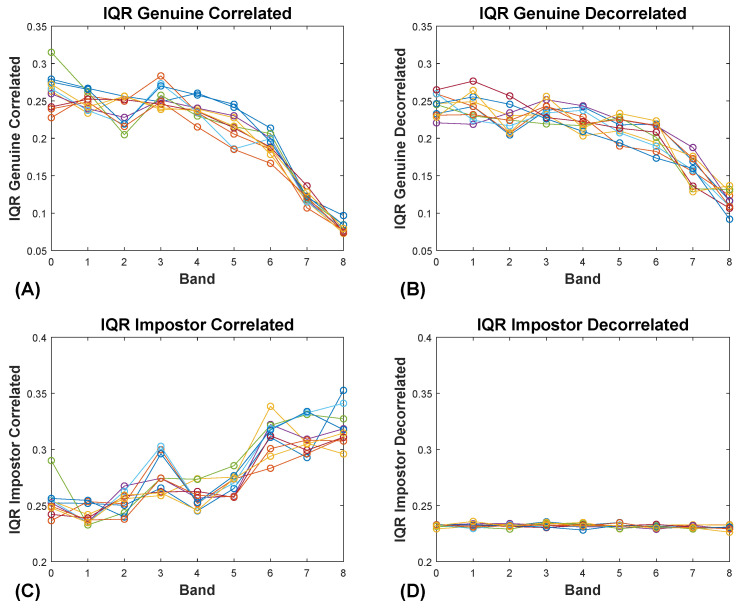
Effects of decorrelation on IQRs. (**A**) Relationship between the IQR of the genuine distribution and ICC Band for the SBA real dataset, prior to decorrelation. (**B**) Relationship between the IQR of the genuine distribution and ICC Band for the SBA real dataset, after decorrelation. (**C**) Relationship between the IQR of the impostor distribution and ICC Band for the SBA real dataset, prior to decorrelation. (**D**) Relationship between the IQR of the impostor distribution and ICC Band for the SBA real dataset, after decorrelation.

In Figure 8A, we see the relationship between ICC band and the IQR of the genuine similarity scores for the original (intercorrelated) SBA dataset. For all the plots in Figure 8, there are 10 lines, each representing a different random selection of 10 of 19 features in each SBA band. In Figure 8B, we see the same data as in Figure 8A after the features within each band have been decorrelated. In Figure 8C, we present the relationship between ICC band and the IQR of the impostor distributions with the raw, intercorrelated data. In Figure 8D we see the same data as in Figure 8C, after the features in each band have been decorrelated. The fact that removing the intercorrelations from the real data reproduces the findings of the synthetic data confirms that this is in fact the explanation for the observed difference.

## 7. General Discussion

Increased temporal persistence improves biometric performance because of specific changes in the similarity score distributions for genuine and impostor samples. We show that, for both synthetic and real data, (1) the median of the distribution of the genuine similarity scores increases with increasing ICC, (2) the median of the distribution of the imposter similarity scores does not change as a function of ICC and (3) the IQR of the similarity scores for the genuine distribution declines with increasing ICC. We refer the reader to Figure 2. A simple comparison of Figure 2B with Figure 2D will make it plain how such changes in the similarity score distributions lead to improved biomeric performance generally and the EER specifically. This is consistent with the view that our synthetic features behave like our real data features and supports the use of these synthetic features to answer additional biometric-related questions.

The key difference between our real data features and our synthetic features has to do with the IQR of the impostor similarity scores, as ICC increases. With synthetic data, the IQR of the impostor distributions does not change with increasing ICC. (Recall that our synthetic features are all, at most, very weakly intercorrelated.) However, with real data features, the IQR of the impostor distributions increase with increasing ICC. We have shown that this difference between the synthetic data and the real data is due to the fact that the degree of intercorrelation among real features increases with increased ICC. This is expected, because higher ICC features have less noise or error variance than lower ICC features. We have shown for real data that increased intercorrelation is associated with increased variance in these real datasets. Furthermore, we have shown that removing these intercorrelations with a decorrelation step removes the differences between the performance of real data and synthetic data.

A further implication of our analysis is that the statement that the features in real datasets are always, or typically, intercorrelated is not comprehensive. Given the availability of decorrelation procedures, it is always the case that real datasets with real intercorrelated features are also, simultaneously, real datasets with completely uncorrelated features.

Although our synthetic features revealed the same pattern as real data features in the main, there was a difference. The difference stemmed from the fact that real features are intercorrelated and our synthetic features are uncorrelated. This suggests the utility of developing synthetic features with intercorrelation patterns similar to real data. The forward (as opposed to inverse) Cholesky transformation can create synthetic datasets given a variance-covariance matrix to emulate. Therefore, the inter-correlation pattern of any real dataset can be emulated in a synthetic dataset easily. The question would remain, which real dataset, or how many different datasets, to emulate? Additionally, what criteria to employ to make this decision? It is not clear to us what the implications of doing this would be on the ICC of such a dataset. This will require more thought and future study.

Interestingly, the relationship between increasing temporal persistence and characteristics of the genuine and impostor similarity scores was noted in our earlier publication [1] (Figure 18 and 19 of that paper). In that paper, based on real biometric datasets, we found a statistically significant increase in the median genuine similarity score as the ICC of the data increased, whereas there was no statistically significant change in the median of the impostor similarity scores (Figure 18 in [1]). We also found a statistically significant decrease in the IQR of the genuine similarity scores for datasets with higher ICC, whereas the change in the IQR for impostor distributions did not change in a statistically significant manner (Figure 19 of [1]). Our theoretical investigations with synthetic data have provided insight into why these phenomena occur and why temporal persistence is so valuable for biometric performance.

## Abbreviations

The following abbreviations are used in this manuscript:

ECG Electrocardiogram EER Equal Error Rater
ICC Intraclass correlation coefficient IQR Interquartile Range
PDF Probability density function
ROC Receiver Operating Characteristic

## Appendix A. R Code to Create Datasets of Synthetic Features

***********************************************
R Code to Create Datasets of Synthetic Features
***********************************************
# Load relevant libraries
library(mise)
library(lme4)
library(matrixStats)
mise() # Clear out variables
# Close all previous connections
closeAllConnections()
n<-1000 # Number of Subjects
k<-10 # Number of Features
s<-2   # Number of Sessions. For~Biometrics, s = 2.
ICC_Target<-0.7 # Target ICC
# Fill feature matrix with zeros
Feature_Matrix <- array(0,dim=c(n,k,s))
# Generate standard normal data for first session of
# all features
Feature_Matrix[,,1] <- rnorm(n*k,0,1)
# Set session 2 measurements equal to the first
# session measurements
Feature_Matrix[,,2] <- Feature_Matrix[,,1]
# At this point, Both session measurements match for
# each feature (for each individual) and ICC = 1.0.
# There is perfect # agreement. We need to add
# noise to features to obtain a lower ICC. The
# noise we add will have a mean of 0 and a
# SD = SD_Noise, calculated as:
SD_Noise<-sqrt((1-ICC_Target)/ICC_Target);
# Add this noise to all features
Feature_Matrix <- Feature_Matrix + rnorm(n*k*s,0,SD_Noise)
# Now we vertically concatenate the sessions
# to get a single matrix of dimension n*s x k
Both_Sessions <- rbind(Feature_Matrix[,,1],Feature_Matrix[,,2])
# Get the column Means
FeatureMeans<-colMeans(Both_Sessions)
cat(sprintf(’Column Means: 1:%0.3f 2:%0.3f 3:%0.3f 4:%0.3f 5:%0.3f 6:%0.3f
7:%0.3f 8:%0.3f 9:%0.3f 10:%0.3f\n’,
FeatureMeans[1],FeatureMeans[2],FeatureMeans[3],FeatureMeans[4],
FeatureMeans[5],FeatureMeans[6],FeatureMeans[7],FeatureMeans[8],
FeatureMeans[9],FeatureMeans[10]))
FeatureSDs<-colSds(Both_Sessions)
# Get the column SDs.
cat(sprintf(’Column SDs: 1:%0.3f 2:%0.3f 3:%0.3f 4:%0.3f 5:%0.3f 6:%0.3f
7:%0.3f 8:%0.3f 9:%0.3f 10:%0.3f\n’,
FeatureSDs[1],FeatureSDs[2],FeatureSDs[3],FeatureSDs[4],
FeatureSDs[5],FeatureSDs[6],FeatureSDs[7],FeatureSDs[8],
FeatureSDs[9],FeatureSDs[10]))
# Create a Subject Number vector
Subject<-c(seq(1:n),seq(1,n))
# Now we create a Session Vector
Session<-vector(mode=’numeric’,length = 0)
Session[1:n]<-1;
Session[(n+1):(n*2)]<-2;
# Horizontally concatenate the Subject Vector,
# the Session vector and the feature data into a
# single dataframe.
Feature_DF <-data.frame(Subject,Session,Both_Sessions)
names(Feature_DF)<-c("Subject","Session","Feat01","Feat02","Feat03",
"Feat04","Feat05","Feat06","Feat07","Feat08", "Feat09","Feat10")
cat(sprintf(’\nWhich directory will the data be written to: %s\n\n’,getwd()))
FeatureFileName<-paste0(’SynthFeatSet_NSess_2_ICC_Targ_’,
as.character(ICC_Target*10),’_NFeat_’,as.character(k),’_NSubs_’,
as.character(n),’.csv’)
#Write out a .csv file with the synthetic features
write.csv(Feature_DF,FeatureFileName,row.names = FALSE)
# Below we illustrate how to compute the ICC using
# variance components.
auto <- function(x) as.data.frame(VarCorr(lmer(x~(1|Feature_DF$Subject) +
(1|Feature_DF$Session),REML = TRUE, na.action=na.omit)))
lmer_results_apply<-apply(Feature_DF[,1:k+2], 2, auto)
ICCs<-vector(mode=’numeric’, length=k)
for (i in 1:k){
  a<-unlist(lmer_results_apply[i])
  VarSub=as.numeric(a[10])
  VarSession=as.numeric(a[11])
  VarErr=as.numeric(a[12])
  TotalVariance<-sum(VarSub,VarSession,VarErr)
  ICCs[i]<-VarSub/TotalVariance
  cat(sprintf(’For Feature %2.2d, VarSubject = %0.3f, VarSession = %0.3f,
  VarErr = %0.3f, TotVar = %0.3f, ICC = %0.3f\n’,
  i,VarSub,VarSession,VarErr,TotalVariance,ICCs[i]))
}
ICC_DF<-data.frame(ICCs)
names(ICC_DF)<-c("ICC")
ICCFileName<-paste0(’SynthFeatSet_ICCs_NSess_2_ICC_Targ_’,
as.character(ICC_Target*10),’_NFeat_’,as.character(k),’_NSubs_’,
as.character(n),’.csv’)
# Write out .csv file with ICCs
write.csv(ICC_DF,ICCFileName,row.names = FALSE)
	

## Appendix B. Variability in Similarity Score and EER Estimates

**Table A1:** Variability in estimates of median genuine and impostor similarity scores. Figure 3A.

	Median of	Minimum of	Maximum of	Median of	Minimum of	Maximum of
	Medians of	Medians of	Medians of	Medians of	Medians of	Medians of
	Genuine	Genuine	Genuine	Impostor	Impostor	Impostor
	Similarity	Similarity	Similarity	Similarity	Similarity	Similarity
Band	Scores	Scores	Scores	Scores	Scores	Scores
0	0.489	0.482	0.493	0.461	0.452	0.466
1	0.542	0.536	0.553	0.461	0.456	0.469
2	0.599	0.592	0.610	0.462	0.454	0.470
3	0.654	0.647	0.658	0.464	0.453	0.470
4	0.708	0.700	0.714	0.459	0.452	0.468
5	0.764	0.758	0.767	0.463	0.457	0.466
6	0.818	0.811	0.822	0.462	0.458	0.472
7	0.868	0.864	0.874	0.458	0.454	0.470
8	0.924	0.920	0.928	0.461	0.450	0.476
9	0.973	0.969	0.981	0.461	0.450	0.471

**Table A2:** Variability in estimates of IQR for genuine and impostor similarity scores. Figure 3B.

	Median of	Minimum of	Maximum of	Median of	Minimum of	Maximum of
	IQRs of	IQRs of	IQRs of	IQRs of	IQRs of	IQRs of
	Genuine	Genuine	Genuine	Impostor	Impostor	Impostor
	Similarity	Similarity	Similarity	Similarity	Similarity	Similarity
Band	Scores	Scores	Scores	Scores	Scores	Scores
0	0.168	0.159	0.175	0.168	0.166	0.171
1	0.164	0.154	0.177	0.168	0.165	0.169
2	0.156	0.148	0.161	0.168	0.165	0.170
3	0.148	0.139	0.153	0.167	0.165	0.170
4	0.134	0.124	0.140	0.168	0.166	0.171
5	0.115	0.109	0.126	0.167	0.166	0.169
6	0.096	0.087	0.103	0.167	0.164	0.168
7	0.072	0.067	0.078	0.169	0.165	0.170
8	0.045	0.042	0.049	0.168	0.163	0.171
9	0.017	0.013	0.019	0.168	0.164	0.171

**Table A3:** Minumum and Maximum EER values for Figure 3, Figure 4 and Figure 5C.

	Minimum	Maximum	Minimum	Maximum	Minimum	Maximum
Band	Synthetic	Synthetic	SBA	SBA	Gait1	Gait
0	43.95	47.20	47.39	49.33		
1	36.10	38.08	41.84	43.59		
2	27.20	29.47	34.90	37.64		
3	20.40	21.98	30.18	31.96		
4	13.70	15.95	25.84	29.02	31.25	34.27
5	8.00	10.00	20.47	22.82	25.00	28.57
6	4.10	5.20	17.45	21.19	19.30	25.44
7	1.50	2.15	10.61	16.14	15.56	18.96
8	0.23	0.60	6.71	10.91	10.51	16.47
9	0.00	0.01				

**Table A4:** Variability in estimates of median genuine and impostor similarity scores. Figure 4A.

	Median of	Minimum of	Maximum of	Median of	Minimum of	Maximum of
	Medians of	Medians of	Medians of	Medians of	Medians of	Medians of
	Genuine	Genuine	Genuine	Impostor	Impostor	Impostor
	Similarity	Similarity	Similarity	Similarity	Similarity	Similarity
Band	Scores	Scores	Scores	Scores	Scores	Scores
0	0.502	0.491	0.515	0.488	0.482	0.490
1	0.554	0.539	0.565	0.488	0.482	0.494
2	0.621	0.607	0.641	0.490	0.486	0.494
3	0.680	0.660	0.694	0.489	0.484	0.493
4	0.735	0.706	0.752	0.487	0.484	0.492
5	0.805	0.785	0.818	0.488	0.484	0.489
6	0.847	0.840	0.855	0.487	0.485	0.491
7	0.899	0.893	0.908	0.487	0.482	0.490
8	0.939	0.934	0.943	0.487	0.478	0.491

**Table A5:** Variability in estimates of IQR for genuine and impostor similarity scores. Figure 4B.

	Median of	Minimum of	Maximum of	Median of	Minimum of	Maximum of
	IQRs of	IQRs of	IQRs of	IQRs of	IQRs of	IQRs of
	Genuine	Genuine	Genuine	Impostor	Impostor	Impostor
	Similarity	Similarity	Similarity	Similarity	Similarity	Similarity
Band	Scores	Scores	Scores	Scores	Scores	Scores
0	0.249	0.252	0.230	0.234	0.285	0.293
1	0.256	0.238	0.220	0.233	0.275	0.257
2	0.244	0.251	0.231	0.238	0.272	0.271
3	0.244	0.265	0.212	0.243	0.261	0.293
4	0.243	0.256	0.228	0.249	0.274	0.267
5	0.219	0.266	0.189	0.253	0.233	0.282
6	0.184	0.313	0.169	0.292	0.206	0.332
7	0.120	0.302	0.104	0.293	0.147	0.357
8	0.078	0.308	0.074	0.299	0.093	0.362

**Table A6:** Variability in estimates of median genuine and impostor similarity scores. Figure 5A.

	Median of	Minimum of	Maximum of	Median of	Minimum of	Maximum of
	Medians of	Medians of	Medians of	Medians of	Medians of	Medians of
	Genuine	Genuine	Genuine	Impostor	Impostor	Impostor
	Similarity	Similarity	Similarity	Similarity	Similarity	Similarity
Band	Scores	Scores	Scores	Scores	Scores	Scores
4	0.758	0.739	0.769	0.493	0.492	0.497
5	0.827	0.800	0.834	0.494	0.491	0.496
6	0.871	0.852	0.892	0.496	0.493	0.499
7	0.902	0.888	0.910	0.492	0.489	0.494
8	0.950	0.941	0.952	0.493	0.491	0.494

**Table A7:** Variability in estimates of IQR for genuine and impostor similarity scores. Figure 5B.

	Median of	Minimum of	Maximum of	Median of	Minimum of	Maximum of
	IQRs of	IQRs of	IQRs of	IQRs of	IQRs of	IQRs of
	Genuine	Genuine	Genuine	Impostor	Impostor	Impostor
	Similarity	Similarity	Similarity	Similarity	Similarity	Similarity
Band	Scores	Scores	Scores	Scores	Scores	Scores
4	0.319	0.280	0.369	0.366	0.340	0.379
5	0.249	0.234	0.277	0.377	0.348	0.407
6	0.190	0.161	0.236	0.414	0.374	0.441
7	0.144	0.119	0.164	0.391	0.367	0.403
8	0.087	0.071	0.107	0.433	0.400	0.478

## References

[1] Friedman L., Nixon M.S., Komogortsev O.V. (2017). Method to assess the temporal persistence of potential biometric features: Application to oculomotor, gait, face and brain structure databases. PLoS ONE.

[2] Friedman L., Stern H.S., Prokopenko V., Djanian S., Griffith H.K., Komogortsev O.V. (2019). Biometric Performance as a Function of Gallery Size. arXiv.

[3] Friedman L., Stern H.S., Komogortsev O.V. (2019). The Linear Relationship between Temporal Persistence, Number of Independent Features and Target EER. arXiv.

[4] Carls J.W., Raines R., Grimaila M., Rogers S. Biometric enhancements: Template aging error score analysis. Proceedings of the 2008 8th IEEE International Conference on Automatic Face & Gesture Recognition.

[5] Fenker S.P., Bowyer K.W. Experimental evidence of a template aging effect in iris biometrics. Proceedings of the IEEE Workshop on Applications of Computer Vision (WACV).

[6] Lanitis A., Tsapatsoulis N. (2011). Quantitative evaluation of the effects of aging on biometric templates. IET Comput. Vis..

[7] Scheidat T., Kümmel K., Vielhauer C. Short Term Template Aging Effects on Biometric Dynamic Handwriting Authentication Performance. Proceedings of the IFIP International Conference on Communications and Multimedia Security.

[8] Fenker S.P., Bowyer K.W. Analysis of template aging in iris biometrics. Proceedings of the 2012 IEEE Computer Society Conference on Computer Vision and Pattern Recognition Workshops.

[9] Fenker S.P., Ortiz E., Bowyer K.W. (2013). Template Aging Phenomenon in Iris Recognition. IEEE Access.

[10] Ortiz E., Bowyer K.W., Flynn P.J. A linear regression analysis of the effects of age related pupil dilation change in iris biometrics. Proceedings of the 2013 IEEE Sixth International Conference on Biometrics: Theory, Applications and Systems (BTAS).

[11] Czajka A. (2014). Influence of iris template aging on recognition reliability. Communications in Computer and Information Science.

[12] Trokielewicz M. Linear regression analysis of template aging in iris biometrics. Proceedings of the 3rd International Workshop on Biometrics and Forensics (IWBF 2015).

[13] Manjani I., Sumerkan H., Flynn P.J., Bowyer K.W. Template aging in 3D and 2D face recognition. Proceedings of the 2016 IEEE 8th International Conference on Biometrics Theory, Applications and Systems (BTAS).

[14] Harvey J., Campbell J., Adler A. (2019). Characterization of Biometric Template Aging in a Multiyear, Multivendor Longitudinal Fingerprint Matching Study. IEEE Trans. Instrum. Meas..

[15] Yue F., Chen X. Template Selection and Update for Biometric Recognition Systems With Nearest Neighbor Classifier. Proceedings of the 2019 Chinese Control Conference (CCC).

[16] Kirchgasser S., Uhl A. Fingerprint Template Ageing Vs. Template Changes Revisited. Proceedings of the 2017 International Conference of the Biometrics Special Interest Group (BIOSIG).

[17] Kirchgasser S., Uhl A., Castillo-Rosado K., Estévez-Bresó D., Rodríguez-Hernández E., Hernández-Palancar J. Fingerprint Template Ageing Revisited—It’s the Quality, Stupid!. Proceedings of the 2018 IEEE 9th International Conference on Biometrics Theory, Applications and Systems (BTAS).

[18] Kirchgasser S., Uhl A. Template ageing in non-minutiae fingerprint recognition. Proceedings of the 2017 5th International Workshop on Biometrics and Forensics (IWBF).

[19] Harvey J., Campbell J., Elliott S., Brockly M., Adler A. Biometric Permanence: Definition and Robust Calculation. Proceedings of the 2017 Annual IEEE International Systems Conference (SysCon).

[20] Labati R.D., Sassi R., Scotti F. ECG biometric recognition: Permanence analysis of QRS signals for 24 hours continuous authentication. Proceedings of the 2013 IEEE International Workshop on Information Forensics and Security (WIFS).

[21] Jain A.K., Ross A., Prabhakar S. (2004). An introduction to biometric recognition. IEEE Trans. Circuits Syst. Video Technol..

[22] Blondet M.V.R., Laszlo S., Jin Z. Assessment of permanence of non-volitional EEG brainwaves as a biometric. Proceedings of the IEEE International Conference on Identity, Security and Behavior Analysis (ISBA 2015).

[23] Tuyls P., Akkermans A.H.M., Kevenaar T.A.M., Schrijen G.J., Bazen A.M., Veldhuis R.N.J., Kanade T., Jain A., Ratha N.K. (2005). Practical Biometric Authentication with Template Protection. Audio- and Video-Based Biometric Person Authentication.

[24] Chen C., Veldhuis R. (2011). Extracting biometric binary strings with minimal area under the FRR curve for the hamming distance classifier. Signal Process..

[25] Chen C., Veldhuis R.N.J., Kevenaar T.A.M., Akkermans A.H.M. (2009). Biometric Quantization through Detection Rate Optimized Bit Allocation. EURASIP J. Adv. Signal Process..

[26] Stampe D. (1993). Heuristic filtering and reliable calibration methods for video-based pupil-tracking systems. Behav. Res. Methods.

[27] Friedman L., Rigas I., Abdulin E., Komogortsev O.V. (2018). A novel evaluation of two related and two independent algorithms for eye movement classification during reading. Behav. Res. Methods.

[28] Rigas I., Friedman L., Komogortsev O. (2018). Study of an extensive set of eye movement features: Extraction methods and statistical analysis. J. Eye Mov. Res..

[29] Shutler J.D., Grant M.G., Nixon M.S., Carter J.N. (2004). On a large sequence-based human gait database. Applications and Science in Soft Computing.

[30] Foster J.P., Nixon M.S., Prugel-Bennett A. (2003). Automatic gait recognition using area-based metrics. Pattern Recognit. Lett..

[31] Kessy A., Lewin A., Strimmer K. (2018). Optimal Whitening and Decorrelation. Am. Stat..

